# Binding Studies on Isolated Porcine Small Intestinal Mucosa and *in vitro* Toxicity Studies Reveal Lack of Effect of *C. perfringens* Beta-Toxin on the Porcine Intestinal Epithelium

**DOI:** 10.3390/toxins7041235

**Published:** 2015-04-09

**Authors:** Simone Roos, Marianne Wyder, Ahmet Candi, Nadine Regenscheit, Christina Nathues, Filip van Immerseel, Horst Posthaus

**Affiliations:** 1 Department of Infectious Diseases and Pathobiology, Institute of Animal Pathology, Vetsuisse Faculty, University of Bern, Bern 3012, Switzerland; E-Mails: simone.roos@vetsuisse.unibe.ch (S.R.); marianne.wyder@vetsuisse.unibe.ch (M.W.); ahmet.candi@vetsuisse.unibe.ch (A.C.); nadine.regenscheit@vetsuisse.unibe.ch (N.R.); 2 Veterinary Public Health Institute, Vetsuisse Faculty, University of Bern, Bern 3012, Switzerland; E-Mail: christina.nathues@vetsuisse.unibe.ch; 3 Department of Pathology, Bacteriology and Avian Medicine, Ghent University, Ghent 9000, Belgium; E-Mail: filip.vanImmerseel@ugent.be

**Keywords:** *Clostridium perfringens* type C, beta-toxin, endothelium, mucosa, epithelium, pathogenesis, porcine

## Abstract

Beta-toxin (CPB) is the essential virulence factor of *C. perfringens* type C causing necrotizing enteritis (NE) in different hosts. Using a pig infection model, we showed that CPB targets small intestinal endothelial cells. Its effect on the porcine intestinal epithelium, however, could not be adequately investigated by this approach. Using porcine neonatal jejunal explants and cryosections, we performed *in situ* binding studies with CPB. We confirmed binding of CPB to endothelial but could not detect binding to epithelial cells. In contrast, the intact epithelial layer inhibited CPB penetration into deeper intestinal layers. CPB failed to induce cytopathic effects in cultured polarized porcine intestinal epithelial cells (IPEC-J2) and primary jejunal epithelial cells. *C. perfringens* type C culture supernatants were toxic for cell cultures. This, however, was not inhibited by CPB neutralization. Our results show that, in the porcine small intestine, CPB primarily targets endothelial cells and does not bind to epithelial cells. An intact intestinal epithelial layer prevents CPB diffusion into underlying tissue and CPB alone does not cause direct damage to intestinal epithelial cells. Additional factors might be involved in the early epithelial damage which is needed for CPB diffusion towards its endothelial targets in the small intestine.

## 1. Introduction

The anaerobic, Gram-positive, spore-forming bacterium *C. perfringens* causes different diseases in humans and animals, such as septicemia, myonecrosis, enterotoxemia, food poisoning and enteritis [1]. Classification into five types is based on the production of four major toxins: Alpha- (CPA), beta- (CPB), epsilon (ETX)- and iota-toxin (ITX) [2]. *C. perfringens* type C produces CPA and CPB; however, additional toxins, such as beta-2 toxin, enterotoxin, perfringolysin and TpeL can be secreted [3,4]. *C. perfringens* type C causes necrotizing enteritis (NE) in newborn animals and in humans [1]. Piglets are most commonly affected and, as in all affected hosts, the hallmark lesion of NE is a severe, segmental, necro-hemorrhagic jejunitis [5]. The exact role of different toxins in the pathogenesis of the disease is not known yet. Experimental studies using genetic approaches and animal models of disease clearly demonstrated that CPB is the essential virulence factor of type C strains [6].

CPB is a soluble 35 kDa monomer protein that is thermo-labile and highly sensitive to degradation by trypsin [4]. It is a member of the beta-barrel pore-forming toxin family and forms oligomeric pores in several susceptible immune cell lines [7,8]. Other studies showed that CPB is required for *C. perfringens*-induced necrotic enteritis in rabbit ileal loops and that purified CPB can produce similar lesions [6]. In this model, lesions were reminiscent of early epithelial damage and thus CPB might also directly act on intestinal epithelial cells. So far, it is however unknown whether epithelial necrosis is caused by a direct effect of the pore-forming toxin on intestinal epithelial cells or indirectly [9,10]. Our recent work showed that endothelial cells are highly susceptible to CPB [11,12] and that pore-formation in endothelial cells rapidly leads to necrotic cell death [13]. In spontaneous disease CPB can be demonstrated in endothelial cells [14,15] and this also correlated to early vascular lesions in an experimental infection model in pigs [16]. Based on these results, we hypothesized that endothelial cells in the small intestine are the primary target for CPB. This however would require that CPB can pass the intestinal epithelial barrier to penetrate into deeper mucosal tissue. A direct toxic effect of CPB on porcine small intestinal epithelial cells is possible and this would facilitate toxicity on endothelial cells. Our hypothesis was however based on analyses of spontaneous end stage lesions [14] and early lesions in experimentally infected piglets [16]. Although we could not demonstrate CPB binding to epithelial cells in these studies, they were not suitable to investigate early epithelial toxicity as they represent stages where tissue necrosis has already begun. Additionally, the piglet intestinal loop model would require a relatively high number of lethal infectious experiments to closely monitor initial interactions of CPB with the small intestinal epithelium. The aim of our study was therefore to evaluate binding of CPB to cells in the porcine small intestinal mucosa and the toxic effect of CPB on cultured porcine small intestinal epithelial cells. To achieve this, we chose two experimental approaches: (I) CPB binding studies using neonatal porcine jejunal cryosections and tissue explants; (II) cellular toxicity studies using a porcine jejunal epithelial cell line (IPEC-J2) and primary porcine jejunal epithelial cells.

## 2. Results

### 2.1. Cellular Binding of CPB in Porcine Jejunal Cryosections

**Figure 1:**
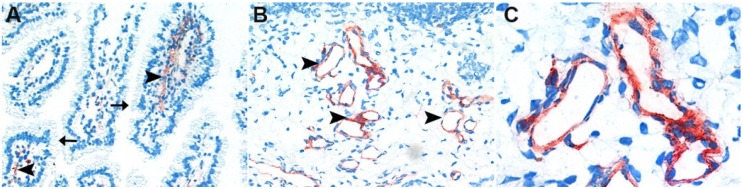
Immunohistochemical localization of CPB (*Clostridium perfringens* beta-toxin) in porcine jejunal cryosections. Cryosections of porcine neonatal jejunum were incubated with *C. perfringens* type C NCTC 3180 supernatant (diluted 1:10 in PBS). CPB was subsequently detected by immunohistochemistry on endothelial cells (arrowheads) in the lamina propria (**A**) and submucosa (**B**), but not on epithelial cells (arrows). (**C**) Magnification of B depicting endothelial CPB signal. Representative pictures from one out of two independent experiments, magnification 400×.

Immunohistochemical stainings of cryosections incubated with supernatants of *C. perfringens* type C NCTC 3180 and type C JF 3721 localized CPB at the endothelium in all layers of the jejunal wall (Figure 1A,B). In contrast, no CPB signal could be demonstrated at the epithelium.

### 2.2. Cellular Binding of CPB in Porcine Jejunal Explants

Histological sections of the porcine neonatal jejunal explants revealed no morphological differences between explants incubated with *C. perfringens* type C or type A supernatants or toxin free control medium. All explants, regardless of the incubation protocol, remained well preserved for the first 4 h of the experiment and afterwards showed early signs of autolysis, such as detachment of epithelial cells. The maximum length of the experiments was therefore set at 6 h of incubation.

**Figure 2:**
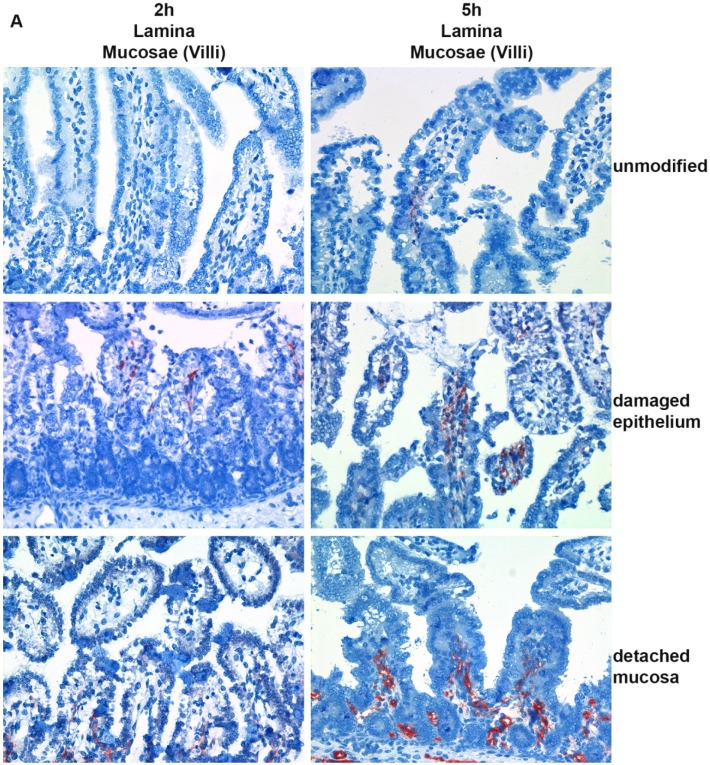

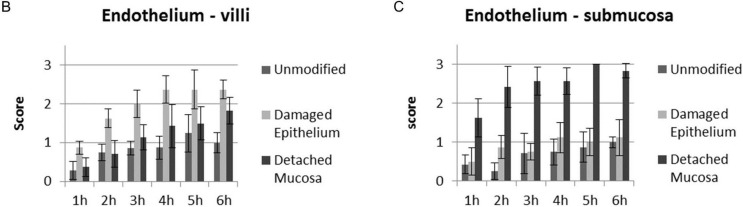
Immunohistochemical localization of CPB in the subepithelial lamina propria of porcine jejunal explants. (**A**) Jejunal explants were either unmodified (unmodified), the epithelium was mechanically damaged (damaged epithelium), or the tunica serosa and tunica muscularis were mechanically detached (detached mucosa). Explants were incubated with *C. perfringens* type C supernatant (JF 3721 diluted 1:10 in RPMI), fixed, and CPB was detected by immunohistochemistry. After 2 h of incubation, mild to moderate CPB signals were detected at endothelial cells close to the damaged epithelium whereas undamaged explants showed no or only very mild signals at 2 h. After 5 h of incubation CPB signals were overall stronger in tissue with a mechanically damaged epithelium. Binding of CPB to the epithelium was not detectable in any explant. Representative pictures from one out of three independent experiments, magnification 400×. (**B**) Mean CPB signal scores and standard deviation at endothelium-villi of eight explants in three independent time course experiments. (**C**) Mean CPB signal scores and standard deviation at endothelium-submucosa (fotographs depicted in Figure S1) of eight explants in three independent time course experiments.

Immunohistochemical staining of all explants incubated with *C. perfringens* type C supernatants showed binding of CPB to the endothelium (Figure 2A). No CPB signal at the epithelium was detectable. In all explants, a weak endothelial signal in vessels and capillaries located directly at the cut margins of the explants was visible from 1 h onwards. These signals were not present in vessels located more than 50 µm from the cut border and were due to diffusion of CPB into the tissue from the cut edges and therefore neglected for our analyses. In unmodified explants, weak endothelial signals in the superficial lamina propria of the villi became evident after 2 h of incubation (Figure 2B); a weak signal in the submucosa of unmodified explants was detectable after 3 h of incubation (Figure 2C). Mechanical damage to the epithelium resulted in the rapid appearance of moderate to strong signals at the endothelium in the villi (Figure 2A,B). Binding to the endothelium in the submucosa was moderate in these preparations (Figure 2C and Figure S1). Detachment of the tunica serosa and tunica muscularis led to the rapid appearance of a stronger CPB signal at the endothelium, especially within the submucosa compared to unmodified explants (Figure 2A,C and Figure S1). Detachment of the tunica serosa and tunica muscularis, however, did not lead to an increased signal intensity at endothelial cells in the villi compared to unmodified explants up to 3 h of incubation (Figure 2A,B). Signal intensities were only increased compared to unmodified explants at 4–6 h of incubation. Pre-incubation of the *C. perfringens* type C supernatants with neutralizing anti-CPB antibodies (mAb-CPB) resulted in complete inhibition of CPB signals in all preparations (Figure S2). Multi-way ANOVA, corrected for animal and time, showed significant differences in scores between the three preparations in the binding of CPB to villous and submucosal endothelium (*p*-values <0.0001). In the case of villous endothelium, all preparation groups showed a significant difference from each other in Tukey-Kramer multiple comparison tests. Explants with a damaged epithelium had a significantly higher chance of exhibiting a moderate or strong signal at the endothelium in villi compared to unmodified explants (OR: 55.3; 95% CI: 15.3–199.0) and explants with a detached mucosa (OR: 4.5; 95% CI: 1.5–13.5) (Figure 2B). For the binding site endothelium-submucosa, detached mucosa explants differed significantly from explants with damaged epithelium or unmodified explants. The chance of detecting a moderate or high signal at the endothelium in the submucosa in explants with a detached mucosa was significantly higher than in unmodified explants (OR: 94.0; 95% CI: 24.9–355.3) and explants with a mechanically damaged epithelium (OR: 59.5; 95% CI: 17.5–202.1) (Figure 2C). There was no significant difference in signal intensities at submucosal endothelia between explants with mechanically damaged epithelium and unmodified explants (OR: 1.6; 95% Cl: 0.49–5.0) (Figure 2C).

These results showed that CPB has a high affinity to endothelial cells in porcine jejunal explants. Despite the lack of epithelial signals in immunohistochemistry, binding of small amounts of CPB to epithelial cells, which could be missed using this technique, cannot be excluded. However, intact small intestinal epithelium seems to act as a diffusion barrier for CPB and prevent tissue penetration of the toxin to reach endothelial cells in the lamina propria and deeper intestinal layers.

### 2.3. Trans Epithelial Electrical Resistance Measurements (TEER)

#### 2.3.1. Apical Exposure of Polarized Porcine Small Intestinal Cells Layers to *C. perfringens* Type C Culture Supernatants and rCPB

**Figure 3:**
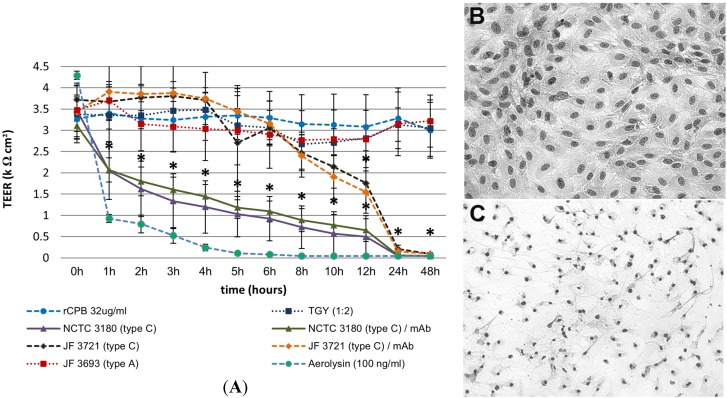
(**A**) Apical exposure of IPEC-J2 cell layers to rCPB does not affect the TEER. IPEC-J2 cells grown on collagen coated Transwells^®^ were incubated with *C. perfringens* supernatants (diluted 1:2 in cell culture medium), TGY (1:2), or rCPB (32 µg/mL) in the apical compartment. Aerolysin used as positive control caused a rapid and sustained drop in TEER. Apical incubation of type C supernatants caused a drop of TEER values within 2 h (NCTC 3180) or 12 h (JF 3721) below 2 kΩ cm^2^. rCPB did not cause a drop of TEER below 2 kΩ cm^2^, similar to type A supernatants and TGY used as controls. Values represent means of three independent experiments with a total of eight separate Transwells^®^. Error bars represent two-fold standard deviation. Asterisks indicate values which differ significantly from the TGY control group. (**B**) Endothelial cells (PAEC) co-cultivated in the basolateral compartment of Transwells^®^ of IPEC-J2 cells apically exposed to rCPB (32 µg/mL) did not exhibit a cytopathic effect. (**C**) Mechanical damage of the IPEC-J2 layer resulted in cytopathic effects in co-cultivated PAEC when the same supernatant as in B was added to the apical compartment. Co-cultivation was performed for 48 h.

To evaluate the effect of *C. perfringens* type C culture supernatants and purified recombinant CPB (rCPB) on the porcine intestinal epithelium, a porcine jejunal epithelial cell line (IPEC-J2) forming a polarized continuous and tight epithelial layer on Transwells^®^ [17] was apically incubated with late log phase culture supernatants of two *C. perfringens* type C strains diluted in cell culture medium. Non-inoculated, sterile TGY and supernatants of a *C. perfringens* type A strain (JF 3693) were used as negative controls. Apical incubation with undiluted sterile TGY reduced the TEER below 1 kΩ cm^2^ but did not do so at a 1:2 dilution. Therefore, all culture supernatants were used at a 1:2 dilution in cell culture medium to achieve maximum CPB concentrations. Similar to TGY, the *C. perfringens* type A supernatant did not have a significant effect on TEER. As a positive control, we used the pore-forming toxin aerolysin (100 ng/mL) which is known to damage epithelial cells [18]. Apical incubation of IPEC-J2 with aerolysin resulted in a significant drop of TEER values below 1 kΩ cm^2^ within 1 h and a subsequent further decline (Figure 3A). The NCTC 3180 supernatant used at a 1:2 dilution (7.5 µg CPB/mL) induced a drop of TEER values below 2 kΩ cm^2^ at 2 h which subsequently further declined (Figure 3A). The supernatant of JF 3721 (2.2 µg CPB/mL) induced a drop in TEER below 2 kΩ cm^2^ starting at 12 h (Figure 3A). Pre-incubation of both type C supernatants with neutralizing mAb-CPB did not reduce this effect. Additionally, both *C. perfringens* type C culture supernatants used at a 1:10 dilution, which contained CPB at a concentration of 1.5 µg/mL (NCTC 3180) and 440 ng/mL (JF 3721), did not cause a drop of TEER below 2 kΩ cm^2^ over 48 h (Figure S3).

Dunn’s Z multiple comparison test revealed a significant difference between TEER values of Transwells^®^ of the TGY control group and Transwells^®^ which had been incubated with aerolysin, neutralized or non-neutralized NCTC 3180 supernatants for 1 h–48 h. TEER values of Transwells^®^ which had been incubated with neutralized JF 3721 supernatant differed significantly from the TGY control group at 12 h–48 h, whereas TEER values of Transwells^®^ incubated with JF 3721 differed significantly at 24 h–48 h. No significant differences were present between Transwells^®^ incubated with type C supernatants (NCTC 3180, JF 3721) and the corresponding supernatants pre-incubated with neutralizing anti-CPB antibodies. No significant differences were detected between TEER values of Transwells^®^ that had been incubated with *C. perfringens* type A and the TGY control group.

Our results using *C. perfringens* type C culture supernatants indicated that CPB was not likely to be the epithelium damaging factor in our experiments. To further evaluate whether CPB was directly toxic to the intestinal epithelium, we incubated polarized IPEC-J2 cells with purified rCPB at a concentration of 32 µg/mL. Recombinant CPB was previously shown to be highly toxic to primary endothelial cells at concentrations of 13 ng/mL, which was in the same range of toxicity as *C. perfringens* type C derived CPB [11]. Even at these approximately 2000-fold higher concentrations, rCPB did not induce a drop of TEER in polarized IPEC-J2 cells (Figure 3A). A lack or the complete loss of cytotoxic activity of rCPB was excluded by incubating PAEC monolayers grown in 96-well plates with apical medium/toxin preparations at the end of each experiment. After 48 h of apical incubation of IPEC-J2, rCPB preparations still induced cell death in PAEC. This effect was inhibited by antibody mediated neutralization of CPB. To evaluate whether rCPB was able to pass the polarized IPEC-J2 layer in the Transwells^®^, PAEC were co-cultivated in the basolateral chamber. Using this approach, no cytopathic effect was observed after 48 h of apical incubation of the IPEC-J2 with rCPB (Figure 3B). Mechanical destruction of the IPEC-J2 layer before apical incubation with rCPB resulted in marked cytotoxicity in co-cultivated PAEC (Figure 3C).

#### 2.3.2. Basolateral Exposure of Polarized Porcine Small Intestinal Cells Layers to *C. perfringens* Type C Culture Supernatants and rCPB

**Figure 4:**
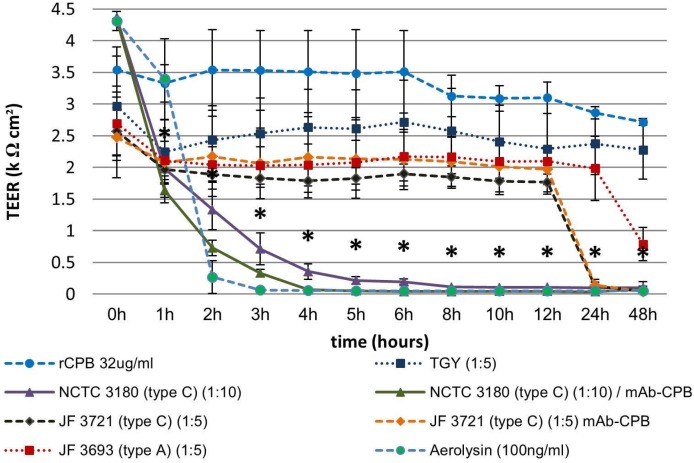
Reduction of TEER upon basolateral exposure of IPEC-J2 cells to *C. perfringens* type C culture supernatants but not rCPB. IPEC-J2 cells were incubated in the basolateral compartment of the Transwell^®^ with the same toxins and supernatants (diluted 1:10 and 1:5) as in Figure 3. Aerolysin and the *C. perfringens* type C supernatant NCTC 3180 (1:10) caused a rapid and sustained drop in TEER. This effect could not be inhibited by neutralization of CPB using mAb-CPB. Supernatants of JF 3721 induced a drop in TEER within 12 h and supernatants of JF 3693 within 48 h. rCPB (32 µg/mL), or TGY (1:5) resulted in a small drop of TEER values, which however never dropped below 2 kΩ cm^2^ and subsequently increased towards the end of the experiment. Values represent means of three independent experiments with a total of eight separate Transwells^®^. Error bars represent two-fold standard deviation. Asterisks indicate values which differ significantly from the TGY control group.

Basolateral exposure of IPEC-J2 cell layers to aerolysin as a positive control resulted in a rapid and complete loss of the TEER of polarized IPEC-J2 layers (Figure 4). TGY did not have an effect on the TEER up to a dilution of 1:5 and the control *C. perfringens* type A supernatant also did not induce a drop in TEER below 2 kΩ cm^2^ at this concentration. Basolateral incubation of polarized IPEC-J2 cells with the supernatant of NCTC 3180 diluted 1:10 in cell culture medium resulted in a rapid decline of TEER. After 1 h, TEER values dropped below 2 kΩ cm^2^ and subsequently declined (Figure 4). Pre-incubation of the supernatants with neutralizing mAb-CPB did not reduce this effect. Basolateral incubation with supernatant of JF 3721 diluted 1:10 in cell culture medium did not induce a drop of TEER values below 2 kΩ cm^2^ (Figure S4), however increasing the concentration to a 1:5 dilution resulted in a significant drop at 24 h with a subsequent further decline (Figure 4). Pre-incubation with mAb-CPB had no effect on this drop in TEER. Recombinant CPB at a concentration of 32 µg/mL did not induce a significant drop in TEER when applied basolaterally. Dunn’s Z multiple comparison tests revealed a significant difference between TEER values of the TGY control group and TEER values of IPEC-J2 grown on Transwells^®^ which had been incubated with aerolysin and the supernatant of NCTC 3180 (1:10) neutralized with mAb-CPB from 1 h–48 h. TEER values of non-neutralized NCTC 3180 differed significantly from 2 h to 48 h from the TGY control group. TEER values of Transwells^®^ incubated with supernatant of JF 3721 neutralized or non-neutralized differed significantly from 24 h to 48 h from the TGY control group. Similar to apical exposure no significant differences were present between Transwells^®^ incubated with type C supernatants (NCTC 3180, JF 3721) and the corresponding neutralized supernatants. Similar to apical exposure experiments, 32 µg/mL CPB in the basolateral chamber of the Transwells^®^ did not reduce the TEER below 2 kΩ cm^2^.

Culture supernatants were considerably more toxic when IPEC-J2 cell layers where exposed basolaterally compared to apical exposure. However, lack of inhibition of the toxic effect by neutralization of CPB and the lack of effect of high rCPB concentrations again indicated that CPB was not essential for this effect.

### 2.4. Exposure of Primary Porcine Small Intestinal Epithelial Cells to C. perfringens Type C Culture Supernatants and rCPB

**Figure 5:**
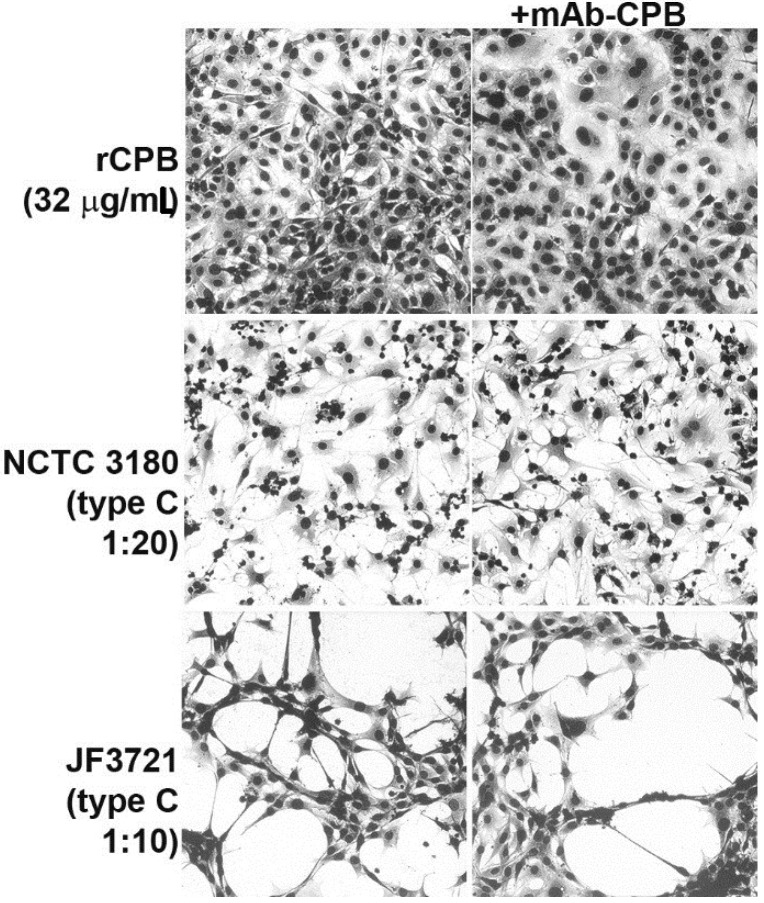
Lack of cytopathic effect of CPB to primary porcine jejunal epithelial cells. rCPB added to the cell culture medium did not cause a cytopathic effect on primary porcine intestinal epithelial cells. Exposure of these cells to *C. perfringens* type C NCTC 3180 and JF 3721 supernatants caused a cytopathic effect, which was not inhibited by pre-incubation with mAb-CPB. Incubation time 48 h.

To exclude that the lack of effect of CPB on polarized IPEC-J2 cell layers was cell line specific, we additionally cultured primary porcine neonatal jejunal epithelial cells. Exposure of primary jejunal epithelial cell monolayers to NCTC 3180 supernatants caused a cytopathic effect at a dilution of 1:20–1:40 (Figure 5). JF 3721 supernatants caused a cytopathic effect at a dilution of 1:2–1:20 (Figure 5). For both type C supernatants, this cytopathic effect was still detectable after pre-incubation with neutralizing antibody mAb-CPB (Figure 5). Recombinant CPB at a concentration of 32 µg/mL (Figure 5) or supernatant of *C. perfringens* type A JF 3693 (data not shown) did not cause a cytopathic effect in primary jejunal epithelial cells.

## 3. Discussion

*C. perfringens* type C causes lethal necrotizing enteritis in animals and humans. We previously showed that endothelial cells are targets for CPB in the porcine small intestine. However, a direct toxic effect of CPB on intestinal epithelial cells was also possible. Using porcine small intestinal mucosal explants and cryosections incubated with *C. perfringens* type C culture supernatants, we now confirmed that CPB preferentially binds to endothelial cells in the porcine small intestinal mucosa but not to epithelial cells. Mechanical damage to the epithelium of intestinal explants led to a more rapid binding of CPB to endothelial cells and overall stronger CPB signals at the endothelial lining of vessels. Our results suggest that undamaged intestinal epithelium acts as a barrier against tissue penetration of CPB. To further evaluate the potential toxic effect of CPB on porcine small intestinal epithelial cells, we investigated the effect of *C. perfringens* type C culture supernatants and purified rCPB on polarized IPEC-J2 cells. These cells form continuous and tight polarized epithelial layers when grown on permeable filters [17] and have frequently been used as a model to investigate the interaction between the porcine intestinal epithelium and bacterial pathogens [19,20]. Apical and basolateral exposure of these cells to medium containing 32 μg/mL rCPB, a concentration which is approximately 2000-fold higher than the reported toxicity to primary porcine endothelial cells [11,12], did not affect the TEER of IPEC-J2 cell layers. This indicates that CPB alone was not toxic to the polarized epithelium. Additionally, we could not detect any toxicity on co-cultivated endothelial cells in the basolateral compartment when IPEC-J2 cells were exposed apically to rCPB, despite the fact that the toxin in the apical chamber was still active on endothelial cells at the end of the experiment. Additionally, rCPB did not affect primary porcine intestinal epithelial cells.

In contrast to purified rCPB, the supernatants of two *C. perfringens* type C strains, one NCTC reference strain and a pathogenic porcine isolate, disrupted the epithelial IPEC-J2 layer when incubated at the apical and basolateral side. The supernatants were generated in TGY bacterial culture medium under conditions optimized for CPB production and were used at the maximum concentration which allowed exclusion of adverse effect by the bacterial culture medium itself. They contained 7.5/1.4 (apical/basolateral exposure to NCTC 3180) or 2.2/0.88 (apical/basolateral exposure to JF 3721) μg/mL CPB, concentrations that were 150–500 fold higher than the toxic concentration for endothelial cells [11]. The same supernatants, albeit at lower concentrations, were also toxic to primary porcine small intestinal epithelial cells. Importantly, the toxic effect of type C supernatants was not reduced when they were pre-incubated with neutralizing anti-CPB antibodies. This indicates that CPB was not responsible for the cytopathic effects.

From our results, we conclude that CPB is non-toxic to polarized IPEC-J2 and primary porcine jejunal epithelial cells at concentrations which are regularly secreted by *C. perfringens* type C isolates under normal growth conditions during late log phase.

In addition, our results show that CPB by itself cannot pass the intact IPEC-J2 polarized epithelial layer under the culture conditions used in our experiments. Together with our data showing decreased tissue penetration in unaltered mucosal explants, this suggests that CPB by itself does not readily pass the intact small intestinal epithelial layer. The most likely explanation for these observations is that intestinal epithelial cells are inert to CPB and that the toxin cannot pass the epithelium either by the paracellular route, which would require damage to tight junctions, or transcellularly. It should be mentioned that the IPEC-J2 cell line grown on Transwells^®^ are unlikely to fully match all physiological properties of the porcine neonatal jejunal epithelium. For example, the small intestinal epithelium of newborn piglets also contains vacuolated fetal-type enterocytes (VFE), which enable the transfer of intestinal contents across the epithelium [21]. Therefore, for example trans- or para-cellular transport of CPB occurring *in vivo* at the jejunal epithelial barrier in newborn piglets cannot be excluded by our results.

Our results further indicate that other secreted factors can induce cytopathic effects in intestinal epithelial cell cultures. In contrast to the effects in cell culture systems, lytic effects of culture supernatants on mucosal explants were not detected. However, as this approach is limited by the onset of post-mortem autolytic effects, it is not well suited to detect such effects. The effect observed in cell culture could be due either to a direct toxicity of secreted factors to epithelial cells or the result of damage to the underlying collagen layers which were used in both cell culture systems. Given the complex pathogenesis of clostridial enteric infections, it is likely that several factors contribute to an initial epithelial damage that, in the case of *C. perfringens* type C infections, enables CPB to penetrate the epithelial barrier and act on endothelial cells. Such effects do not necessarily have to be related to direct toxicity by a particular toxin or enzyme. Recently several publications suggested synergistic effects of secreted *C. perfringens* toxins in gas gangrene [22] but also enteric infections. Verherstraeten *et al.* [23] reported on synergistic effects of PFO and CPA in bovine *C. perfringens* type A induced necrohemorrhagic enteritis and Ma *et al.* [24] demonstrated a synergistic effect of enterotoxin (CPE) and CPB from pathogenic human *C. perfringens* type C isolates in rabbit ileal loops. Our strains did not carry the *cpe* gene; thus, CPE as a contributing factor can be excluded in our experiments.

In limited evaluations we were not able to correlate the toxic effects to the level of alpha-toxin (CPA), perfringolysin (PFO) and collagenase. We did not investigate the contribution of further toxins known to be produced by *C. perfringens* type C strains such as Tpel or beta-2-toxin. Further studies including exposure to purified toxins or enzymes and toxin gene knockout mutants of *C. perfringens* would be needed to identify particular virulence factors which are involved in intestinal epithelial damage. In addition, genome and plasmid sequencing of *C. perfringens* type C strains and analyses of culture supernatants might reveal additional virulence factors in the future.

Interestingly, purified CPB alone injected into rabbit ileal loops rapidly causes necro-hemorrhagic lesions [25] which raises the question whether CPB alone could act on epithelial cells. However, it should be taken into account that ileal loop models in any species represent an unphysiological condition in the intestine. Disruption of normal intestinal motility and passage will unequivocally lead to changes in the microflora and could have rapid functional effects on the small intestinal mucosal barrier, which might not be depicted by morphological studies. In addition, the ligations themselves will induce pressure and ischemic damage to the intestinal wall and also epithelium which, at least locally, will disrupt the epithelial barrier of the intestine. In this respect, it is noteworthy that control loops in the experiments of Ma *et al.* [24] inoculated with culture medium alone histologically also showed very minor lesions. These might be sufficient to allow CPB to pass the intestinal epithelium and target susceptible cells in the lamina propria and deeper layers of the mucosa. Another reason for the discrepancy between our results and the effects of purified CPB in rabbit intestinal loops and a recently developed mouse oral and duodenal inoculation model [26] could be different susceptibilities of cells in rabbits, mice and pigs to CPB. CPB is a beta-barrel pore forming toxin [27]. These toxins are secreted as monomers and bind to susceptible target cells via specific receptors, where they oligomerize and finally form a membrane spanning pore [27]. Cellular receptors of CPB and therefore their distribution on different cells and in different species are still unknown. Beta-toxin has been shown to form oligomeric pores in several human immune cell lines [7,8] and endothelial cells [28]. In immune cell lines and endothelial cells [13] this leads to rapid cell death. Many other cells, such as Hela-, Vero-, CHO-, MDCK-, Cos-7-, P-815, PC12 and fibroblasts cells, were reported to be insensitive to CPB [7,11,12,29,30]; however, literature comparing cellular susceptibilities to CPB is rare. The most likely explanation for this effect is the differential expression of CPB receptors on these cells. Similarly, receptors could be differentially expressed between different species and therefore the different models used to study the mode of action of CPB might not directly be comparable.

## 4. Conclusions

**Figure 6:**
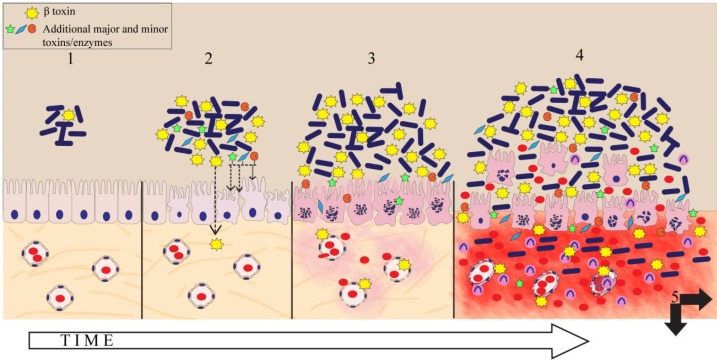
Hypothesis on key events of *C. perfringens* type C induced necrotizing enteritis in pigs triggered by CPB: Enteric disease most likely results from a coordinated interplay between different events and factors. (1) The disease starts with colonization and rapid proliferation of *C. perfringens* type C. (2) Initial epithelial damage/irritation could be caused by secreted toxins and/or be due to the altered luminal environment. This enables CPB to pass the epithelial barrier and diffuse into the lamina propria. 3. Due to the high susceptibility of endothelial cells to CPB, even small amounts of CPB reaching the endothelium result in local vascular leakage. (3) This increases epithelial damage and leakage of blood and tissue components into the intestinal lumen which favors clostridial growth and toxin production [31]. (4) Due to the high proliferative capacity of *C. perfringens* under these local environmental conditions, a vicious cycle of CPB induced hemorrhage, accelerated clostridial growth and toxin secretion develops. (5) This would explain the rapidly progressing hemorrhage and necrosis of the small intestine, the hallmark lesion of *C. perfringens* type C enteritis. In addition, toxins from the intestinal lumen can be absorbed into the systemic circulation and cause enterotoxemia. Toxicity of CPB to immune cells [8] could additionally contribute to the disease at various stages. Not depicted in our hypothesis model is the mucous layer on the epithelium. Currently, we have no knowledge about interactions of *C. perfringens* type C with this important protective layer in the small intestine.

In conclusion, we confirmed that in the porcine small intestine, CPB preferentially binds to endothelial cells and that it does not appear to bind to small intestinal epithelial cells nor does it have a direct toxic effect on the porcine small intestinal epithelium. This further supports the hypothesis that local damage to the intestinal vasculature mediated by CPB is a key trigger for the development of the disease. Additional secreted factors but also intestinal environmental changes occurring during the onset of *C. perfringens* type C enteritis are potentially involved in primary epithelial damage, which is required for the diffusion of CPB through the intestinal epithelial barrier to reach its main target: endothelial cells. Together with results from recent studies by other groups, we can hypothesize on several key events in the pathogenesis of *C. perfringens* type C enteritis (Figure 6). Further research will be needed on the complex interaction of *C. perfringens* with the intestinal microenvironment, the mucosa and associated immune system, epithelium damaging factors and receptor identification of different toxins in order to gradually complete our picture of the complex pathogenesis of clostridial enteric diseases.

## 5. Experimental Section

### 5.1. Production of C. perfringens Culture Supernatants, Recombinant CPB and Aerolysin

*C. perfringens* strains used were: The porcine *C. perfringens* type C strain JF 3721 [11] (genotype: *cpa*, *cpb*, *cpb2*, *pfo*, *tpel*) isolated from an outbreak of NE in 2006, the type C reference strain NCTC 3180 (sheep peritonitis isolate; genotype: *cpa*, *cpb*, *cpb2*, *pfo*, *tpel*) and the porcine *C. perfringens* type A strain JF 3693 [11] (porcine isolate; genotype *cpa*, *cpb2*, *pfo*) were grown anaerobically on blood agar plates. Overnight anaerobic cultures were produced using liquid TGY broth (trypsin glucose yeast extract broth, 3% tryptic soy broth (BD 286220), 2% d(+) glucose (Merck Millipore, Darmstadt, Germany), 1% yeast extract (BD 212750), 0.1% l-cysteine hydrochloride (Sigma-Aldrich, St. Louis, MO, USA) as described previously [11]. Aliquots of these cultures were transferred to 50 mL TGY and cultivated until they reached late-log phase. The cultures were then chilled on ice and centrifuged (Hettich, Rotanta 460 R, 2602 g, 4 °C, 20 min). Supernatants were sterile filtered using 0.22 µm Millex GV filter units (Merck Millipore, Darmstadt, Germany) and stored on ice until use in the experiments within the next three hours. The amount of CPA in the supernatant was quantified using an Alpha Toxin Elisa Kit (Bio-X Diagnostics, Jemelle, Belgium) with *C. perfringens* phospholipase C (Sigma-Aldrich, St. Louis, MO, USA) as standard. Collagenase activity was quantified using the EnzChek^®^ Gelatinase/Collagenase Assay Kit (Molecular Probes^®^, Lifetechnologies™, Carlsbad, CA, USA) according to the manufacturer’s recommendation. Western-blots to quantify CPB in culture supernatants were carried out as described [11]. Perfringolysin activity was measured using a dilution horse erythrocyte hemolysis assay as described previously [32,33]. Toxin concentrations of the *C. perfringens* strains were: JF 3721: 4.4 µg/mL CPB, 24 µU/mL CPA, 3343.56 U/mL collagenase activity, 4 PFO activity Log_2_ (titer); NCTC 3180: 15 µg/mL CPB, 110 µU/mL CPA, 5179.04 U/mL collagenase activity, 5.75 PFO activity Log_2_ (titer); JF 3693: 33 µU/mL CPA, 4041.69 U/mL collagenase activity, 4.25 PFO activity Log_2_ (titer). Expression and purification of rCPB was performed as described [11]. Cytotoxic activity of rCPB and *C. perfringens* type C culture supernatants to primary porcine endothelial cells (PAEC) was verified before every experiment and was similar to our previous study, where diluted *C. perfringens* type C supernatants containing 15 ng/mL CPB and rCPB concentrations of 13 ng/mL were still toxic to PAEC [11]. Proaerolysin was kindly provided by F. G. van der Goot (Global Health Institute, Ecole Polytechnique Federale de Lausanne, Lausanne, Switzerland) and activated as described [34].

### 5.2. Jejunal Explants, Histology, Immunohistochemistry

All animal procedures were approved by the Bernese Cantonal Veterinary Office and the ethical committee of the Faculty of Veterinary Medicine, Ghent University. Four neonatal, colostrum deprived piglets were deeply anaesthetized (Stresnil^®^ 0.05 mL/kg, Sanochemia Pharmazeutika AG, Neufeld, Austria; Ketanerkon^®^ 0.15 mL/kg, Streuli Pharma AG, Uznach, Switzerland; Morphasol 0.03 mL/kg, Dr. E. Gräub AG, Bern, Switzerland) and euthanized by exsanguination. The jejunum was immediately harvested and transferred to PBS containing 1× antibiotic/antimycotic (Ab/Am, Gibco^®^, Lifetechnologies™, Carlsbad, CA, USA). Sections of 10 cm were opened and flushed several times. The samples were transferred to RPMI 1640 (Gibco^®^, Lifetechnologies™, Carlsbad, CA, USA; Catalog number 21875) containing 1 × Ab/Am and 150 µg/mL trypsin inhibitor (TI, Trypsin inhibitor from Glycine max (soybean), Sigma-Aldrich, St. Louis, MO, USA). Intestinal samples were prepared in three different ways: Explants remained unmodified (unmodified), the epithelial layer was damaged by scraping with a scalpel blade (damaged epithelium), or the tunica serosa and tunica muscularis were mechanically detached by pulling with forceps, with only the submucosa and mucosa remaining (detached mucosa). The explants were then cut in approximately 1 cm sections and transferred to 6-well-plates. Each well contained 6 mL of *C. perfringens* culture supernatant diluted 1:10 in RPMI (1 × Ab/Am, 150 µg/mL TI). TGY diluted 1:10 in the same medium and medium without additives were used as controls. The explants were incubated at a temperature of 37 °C. At different time points (1, 2, 3, 4, 5, 6 h) explants were placed in 10% buffered formalin and fixed for 24 h at room temperature (RT). Tissue sections were then embedded in paraffin and routinely processed for histology, cut into 5 µm sections and stained with hematoxylin and eosin (HE). Immunohistochemical analyses were performed using monoclonal anti-CPB antibodies (mAb-CPB 10 A2, Centre for Veterinary Biologics, Ames, IA, USA) as described previously [15]. Each experiment using tissue explants was performed independently three times on tissue from different piglets. Binding of CPB to endothelial cells within villi or the submucosa and/or epithelial cells was determined by light microscopy and graded according to the following grading scheme: 0 = no signal, 1 = weak signal, only a small number of vessels (up to 30%) were affected, 2 = moderate signal, a moderate number of vessels (30%–70% of vessels) were affected 3 = strong signal, most vessels (more than 70%) were affected.

### 5.3. Binding Studies in Intestinal Cryosections

Jejunal tissue of a freshly euthanized colostrum deprived neonatal piglet was flushed several times in PBS, embedded in Tissue Tek O.C.T Compound. (Sakura Finetec USA, Torrance, CA, USA) and frozen in liquid nitrogen. Blocks were stored at −80 °C and cut at a temperature of −20 °C into 4.5 µm sections using a cryomicrotome. Tissue sections were mounted on Superfrost^®^ plus glass slides (Thermo Scientific, Waltham, MA, USA) and were stored at −20 °C. Tissue sections were incubated for 15 h at 4 °C with supernatant of *C. perfringens* strains NCTC 3180, JF 3721, JF 3693 diluted 1:10 in PBS. No fixation was performed prior to immunostaining. Sections were washed with PBS and immunohistochemical detection of CPB was performed using the same protocol as for paraffin sections [15]. The experiment was performed independently twice on tissue sections from two different animals.

### 5.4. Antibody Neutralization Experiments

*C. perfringens* type C culture supernatants were pre-incubated with neutralizing mAb-CPB for 1 h as previously described [11].

### 5.5. Cell Cultures

Primary porcine aortic endothelial cells (PAEC) were obtained and grown as described [11]. The intestinal porcine jejunal cell line (IPEC-J2) [19] was grown in cell culture medium (DMEM/Ham’s F12, Gibco; 2× Insulin-Transferrin-Selenium (Gibco^®^, Lifetechnologies™, Carlsbad, CA, USA); 5% fetal calf serum (FCS); 1 × Ab/Am; 1 × l-Glutamine (Gibco^®^, Lifetechnologies™, Carlsbad, CA, USA). For the isolation of primary porcine intestinal epithelial cells 20 cm segments of jejunum were obtained from a freshly euthanized piglet and transferred to a petri dish containing Hanks’ balanced salt solution (HBSS, Gibco; 5 × Ab/Am). Segments were rinsed with HBSS 5 × Ab/Am and opened longitudinally. The mucosa was mechanically detached from the tunica muscularis and tunica serosa and cut into 3 mm pieces. These were washed with HBSS 5 × Ab/Am, transferred to 15 mL HBSS 1 × Ab/Am containing 10 µg/mL Collagenase/Dispase (Roche Diagnostics GmbH, Mannheim, Germany) and incubated for 60 min at 37 °C under gentle agitation. Finally, the tissue was dissociated by incubation in trypsin EDTA, sieved using BD Falcon^®^ cell strainers (70 µm, Fisher Scientific, Lucens, Switzerland, REF 352350) and centrifuged (131 g, RT, 15 min). The cell pellets were resuspended in medium (DMEM; 5% FCS; 1× Insulin-Transferrin-Selenium; 5 ng/mL epidermal growth factor (EGF, Invitrogen™, Lifetechnologies™, Carlsbad, CA, USA); 1 × Ab/Am) and seeded at a density of 3 × 10^4^/cm^2^ onto collagen coated (4 µg collagen (bovine collagen 1, Cultrex^®^, Trevigen, Gaithersburg, Maryland, USA)/cm^2^) 6-well plates. Medium was replaced after 24 h and then every 3 days. Initially isolated cells reached confluency after 7–14 days. Epithelial origin of primary cells was confirmed using immunofluorescence for cytokeratin. These primary porcine intestinal epithelial cells were then seeded on collagen coated 8-well Nun™ Lab Tek™ (Thermo Scientific, Waltham, MA, USA), grown for 5 days until confluency and fixed with acetone for 10 min at −20 °C. Cells were incubated 1 h RT with monoclonal anti-cytokeratin 18 antibody (Sigma-Aldrich, St. Louis, MO, USA) followed by AlexaFluor goat-anti mouse IgG (Lifetechnologies™, Carlsbad, CA, USA). All washing steps were performed with PBS pH 7.5, three times for 5 min.

### 5.6. Transepithelial Resistance Measurements

IPEC-J2 were seeded onto Transwell^®^ (Sigma-Aldrich, St. Louis, MO, USA) membranes (6.5 mm Transwell^®^-COL Collagen-Coated 0.4 pore PTFE Membrane Insert) with a density of 10^5^/Transwell^®^ and kept in culture for 8–14 days, until trans epithelial electrical resistance (TEER) reached a minimum of 2 kΩ/cm^2^. These TEER values are indicative of a continuous epithelial layer with tight junction formation [35]. TEER was measured using the EVOM^2^ Voltohmmeter (World Precision Instruments, Sarasota, FL, USA). Diluted rCPB, *C. perfringens* culture supernatants or control medium was added either in the apical (containing 200 μL fluid) or the basolateral chamber (containing 1 mL fluid) of the Transwell^®^. *C. perfringens* culture supernatants diluted 1:2 for apical incubation and 1:5 or 1:10 for basal incubation in cell culture medium were used. Higher concentrations of supernatants were not used because at these concentrations non-inoculated (sterile) TGY started to affect TEER values. Confluent monolayers of PAEC grown on fibronectin (1 µg fibronectin (Fibronectin, plasma, purified human, Calbiochem)/cm^2^) coated glass cover slips were placed in the basolateral compartment (24-well cell culture plates) of Transwell^®^ for co-cultivation experiments. To test for residual CPB activity in medium from the apical chamber at the end of the experiments, twofold dilution steps of the medium in this chamber were added to confluent PAEC grown in 96-well plates. Cytopathic effects in PAEC were evaluated after 24 h of incubation by light microscopy.

### 5.7. Cytotoxicity in Primary Porcine Jejunal Epithelial Cells

For detection of cytopathic effects in primary intestinal epithelial cells, two-fold dilution steps of rCPB and supernatants of *C. perfringens* strains were added to confluent monolayers grown in collagen coated 96-well plates. Cytopathic effects were evaluated after 24 h of incubation by light microscopy.

### 5.8. Statistical Analyses

Statistical analyses were carried out using NCSS 9 Data software (NCSS, LLC, Kaysville, UT, USA). Multi-way ANOVA was performed to assess differences in signal intensity between the three different preparation groups (unmodified, damaged epithelium, detached mucosa) of the explant study. Values were corrected for time and animal, at different locations of the endothelium (*i.e.*, in villi and submucosa) and epithelium separately. Identification of different groups was done via Tukey-Kramer multiple comparison tests. Since not all model assumptions were met (e.g., the dependent variable was not truly continuous), a multivariable logistic regression analysis was performed to confirm results of the previous analyses. Therefore, the dependent variable was recoded into two categories (no to weak signal (0,1); moderate to strong signal (2,3)) whereas the same independent variables were used. Differences in signal strength (binary variable) between the binding sites endothelium-villi and endothelium-submucosa for unmodified explants were assessed via chi^2^ test. Kruskal-Wallis tests were performed to assess differences in TEER values between the different incubation groups for each point in time separately. Dunn’s Z multiple comparison tests were used to identify significant differences between different incubation groups.

## Supplementary Materials

Supplementary materials can be accessed at: http://www.mdpi.com/2072-6651/7/4/1235/s1.

## References

[1] Songer J.G. (1996). Clostridial enteric diseases of domestic animals. Clin. Microbiol. Rev..

[2] Petit L., Gibert M., Popoff M.R. (1999). *Clostridium perfringens*: Toxinotype and genotype. Trends Microbiol..

[3] Amimoto K., Noro T., Oishi E., Shimizu M. (2007). A novel toxin homologous to large clostridial cytotoxins found in culture supernatant of *Clostridium perfringens* type C. Microbiology.

[4] Popoff M.R., Bouvet P. (2009). Clostridial toxins. Future Microbiol..

[5] Jäggi M., Wollschlager N., Abril C., Albini S., Brachelente C., Wyder M., Posthaus H. (2009). Retrospective study on necrotizing enteritis in piglets in switzerland. Schweiz. Arch. Tierheilkd..

[6] Sayeed S., Uzal F.A., Fisher D.J., Saputo J., Vidal J.E., Chen Y., Gupta P., Rood J.I., McClane B.A. (2008). Beta toxin is essential for the intestinal virulence of *Clostridium perfringens* type C disease isolate cn3685 in a rabbit ileal loop model. Mol. Microbiol..

[7] Nagahama M., Hayashi S., Morimitsu S., Sakurai J. (2003). Biological activities and pore formation of *Clostridium perfringens* beta toxin in hl 60 cells. J. Biol. Chem..

[8] Nagahama M., Shibutani M., Seike S., Yonezaki M., Takagishi T., Oda M., Kobayashi K., Sakurai J. (2013). The p38 mapk and jnk pathways protect host cells against *Clostridium perfringens* beta-toxin. Inf. Immun..

[9] Uzal F.A., McClane B.A. (2011). Recent progress in understanding the pathogenesis of *Clostridium perfringens* type C infections. Vet. Microbiol..

[10] Los F.C., Randis T.M., Aroian R.V., Ratner A.J. (2013). Role of pore-forming toxins in bacterial infectious diseases. Microbiol .Mol. Biol. Rev..

[11] Gurtner C., Popescu F., Wyder M., Sutter E., Zeeh F., Frey J., von Schubert C., Posthaus H. (2010). Rapid cytopathic effects of *Clostridium perfringens* beta-toxin on porcine endothelial cells. Inf. Immun..

[12] Popescu F., Wyder M., Gurtner C., Frey J., Cooke R.A., Greenhill A.R., Posthaus H. (2011). Susceptibility of primary human endothelial cells to *C. perfringens* beta-toxin suggesting similar pathogenesis in human and porcine necrotizing enteritis. Vet. Microbiol..

[13] Autheman D., Wyder M., Popoff M., D’Herde K., Christen S., Posthaus H. (2013). *Clostridium perfringens* beta-toxin induces necrostatin-inhibitable, calpain-dependent necrosis in primary porcine endothelial cells. PLoS One.

[14] Miclard J., Jaggi M., Sutter E., Wyder M., Grabscheid B., Posthaus H. (2009). *Clostridium perfringens* β-toxin targets endothelial cells in necrotizing enteritis in piglets. Vet. Microbiol..

[15] Miclard J., van Baarlen J., Wyder M., Grabscheid B., Posthaus H. (2009). *Clostridium perfringens* beta-toxin binding to vascular endothelial cells in a human case of enteritis necroticans. J. Med. Microbiol..

[16] Schumacher V.L., Martel A., Pasmans F., van Immerseel F., Posthaus H. (2013). Endothelial binding of beta toxin to small intestinal mucosal endothelial cells in early stages of experimentally induced *Clostridium perfringens* type C enteritis in pigs. Vet. Pathol..

[17] Schierack P., Nordhoff M., Pollmann M., Weyrauch K.D., Amasheh S., Lodemann U., Jores J., Tachu B., Kleta S., Blikslager A. (2006). Characterization of a porcine intestinal epithelial cell line for *in vitro* studies of microbial pathogenesis in swine. Histochem. Cell Biol..

[18] Abrami L., Fivaz M., Glauser P.E., Sugimoto N., Zurzolo C., van der Goot F.G. (2003). Sensitivity of polarized epithelial cells to the pore-forming toxin aerolysin. Inf. Immun..

[19] Boyen F., Pasmans F., van Immerseel F., Donne E., Morgan E., Ducatelle R., Haesebrouck F. (2009). Porcine *in vitro* and *in vivo* models to assess the virulence of salmonella enterica serovar typhimurium for pigs. Lab. Anim..

[20] Brosnahan A.J., Brown D.R. (2012). Porcine ipec-j2 intestinal epithelial cells in microbiological investigations. Vet. Microbiol..

[21] Skrzypek T., Valverde Piedra J.L., Skrzypek H., Kazimierczak W., Biernat M., Zabielski R. (2007). Gradual disappearance of vacuolated enterocytes in the small intestine of neonatal piglets. J. Physiol. Pharmacol..

[22] Awad M.M., Ellemor D.M., Boyd R.L., Emmins J.J., Rood J.I. (2001). Synergistic effects of alpha-toxin and Perfringolysin O in *Clostridium perfringens*-mediated gas gangrene. Inf. Immun..

[23] Verherstraeten S., Goossens E., Valgaeren B., Pardon B., Timbermont L., Vermeulen K., Schauvliege S., Haesebrouck F., Ducatelle R., Deprez P. (2013). The synergistic necrohemorrhagic action of *Clostridium perfringens* perfringolysin and alpha toxin in the bovine intestine and against bovine endothelial cells. Vet. Res..

[24] Ma M., Gurjar A., Theoret J.R., Garcia J.P., Beingesser J., Freedman J.C., Fisher D.J., McClane B.A., Uzal F.A. (2014). Synergistic effects of *Clostridium perfringens* enterotoxin and beta toxin in rabbit small intestinal loops. Inf. Immun..

[25] Vidal J.E., McClane B.A., Saputo J., Parker J., Uzal F.A. (2008). Effects of *Clostridium perfringens* beta-toxin on the rabbit small intestine and colon. Inf. Immun..

[26] Uzal F.A., Saputo J., Sayeed S., Vidal J.E., Fisher D.J., Poon R., Adams V., Fernandez-Miyakawa M.E., Rood J.I., McClane B.A. (2009). Development and application of new mouse models to study the pathogenesis of *Clostridium perfringens* type C enterotoxemias. Inf. Immun..

[27] Popoff M.R. (2014). Clostridial pore-forming toxins: Powerful virulence factors. Anaerobe.

[28] Steinthorsdottir V., Halldorsson H., Andresson O.S. (2000). *Clostridium perfringens* beta-toxin forms multimeric transmembrane pores in human endothelial cells. Microb. Pathog..

[29] Manich M., Knapp O., Gibert M., Maier E., Jolivet-Reynaud C., Geny B., Benz R., Popoff M.R. (2008). *Clostridium perfringens* delta toxin is sequence related to beta toxin, netb, and staphylococcus pore-forming toxins, but shows functional differences. PLoS One.

[30] Shatursky O., Bayles R., Rogers M., Jost B.H., Songer J.G., Tweten R.K. (2000). *Clostridium perfringens* beta-toxin forms potential-dependent, cation-selective channels in lipid bilayers. Inf. Immun..

[31] Vidal J.E., Ohtani K., Shimizu T., McClane B.A. (2009). Contact with enterocyte-like caco-2 cells induces rapid upregulation of toxin production by *Clostridium perfringens* type C isolates. Cell. Microbiol..

[32] Fisher D.J., Fernandez-Miyakawa M.E., Sayeed S., Poon R., Adams V., Rood J.I., Uzal F.A., McClane B.A. (2006). Dissecting the contributions of *Clostridium perfringens* type C toxins to lethality in the mouse intravenous injection model. Inf. Immun..

[33] Stevens D.L., Mitten J., Henry C. (1987). Effects of alpha and theta toxins from *Clostridium perfringens* on human polymorphonuclear leukocytes. J. Inf. Dis..

[34] Krause K.H., Fivaz M., Monod A., van der Goot F.G. (1998). Aerolysin induces *g*-protein activation and Ca^2+^ release from intracellular stores in human granulocytes. J. Biol. Chem..

[35] Fromter E., Diamond J. (1972). Route of passive ion permeation in epithelia. Nat. New Biol..

