# Supplementary material for: Binding Studies on Isolated Porcine Small Intestinal Mucosa and in vitro Toxicity Studies Reveal Lack of Effect of C. perfringens Beta-Toxin on the Porcine Intestinal Epithelium
Source: Toxins (Basel). 2015 Apr 9;7(4):1235–52. doi: 10.3390/toxins7041235 (PMC4417965; doi:10.3390/toxins7041235)
Supplement: Supplementary file 1 [file toxins-07-01235-s001.pdf]

# Supplementary Information

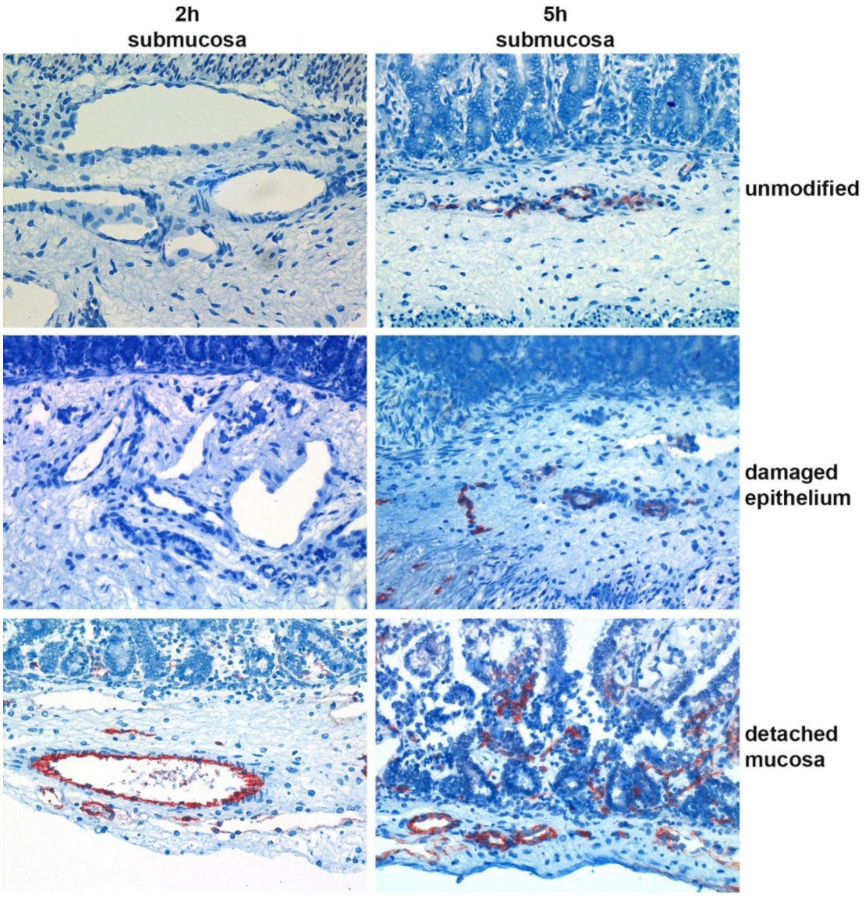


**Figure S1.** Immunohistochemical localization of CPB in the submucosa of porcine jejunal explants. Jejunal explants were either unmodified (unmodified), the epithelium was mechanically damaged (damaged epithelium), or the tunica serosa and tunica muscularis were mechanically detached (detached mucosa). Explants were incubated with *C. perfringens* type C supernatant (JF 3721 diluted 1:10 in RPMI), fixed, and CPB was detected by immunohistochemistry. After 2 h of incubation, CPB signals were detected at endothelial of the submucosa in explants consisting of detached mucosa (underlying tunica muscularis and serosa mechanically detached). After 5 h of incubation, CPB signals were overall stronger and also appeared in unmodified explants or explants with a mechanically damaged epithelium. Representative pictures from one out of three independent experiments, magnification 400×.

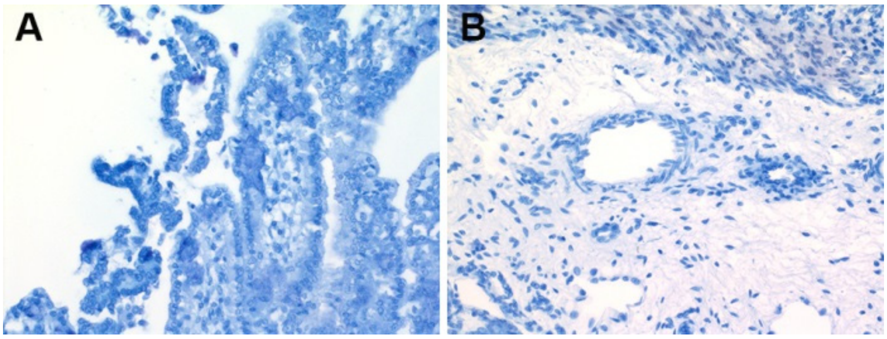


**Figure S2.** Preincubation of *C. perfringens* type C supernatant with mAB-CPB resulted in complete inhibition of signals in all tissue layers (A lamina propria; B submucosa) in all preparations. Representative pictures from one experiment, unmodified explant incubated with neutralized supernatant of NCTC 3180 for 4 h, 400×.

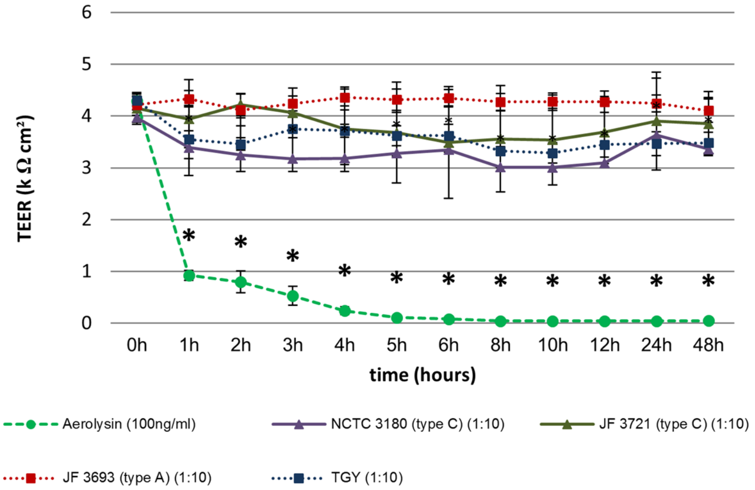


**Figure S3.** Apical exposure of IPEC-J2 to *C. perfringens* type C and type A supernatants diluted 1:10 in cell culture medium did not alter TEER values. Type C strains contained CPB at a concentration of 1.5 µg/mL (NCTC 3180) and 440 ng/mL (JF 3721).

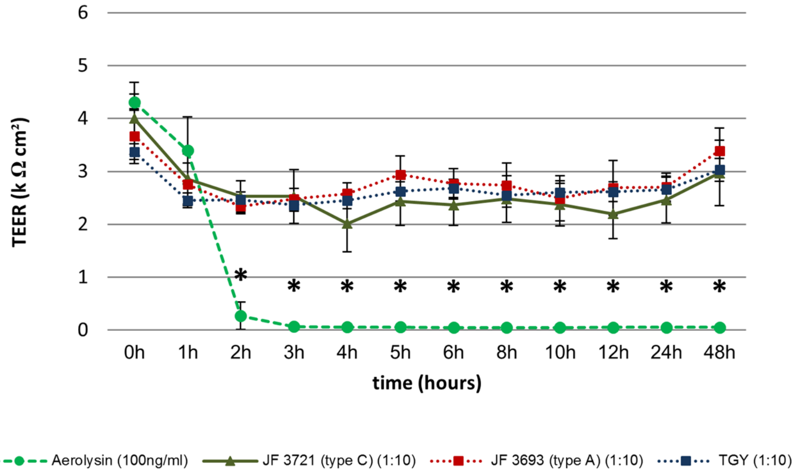


**Figure S4.** Basolateral exposure of IPEC-J2 to the supernatants of JF 3721 and JF 3693 diluted 1:10 in cell culture medium did not induce a drop of TEER values below 2 k Ω $cm^2$. Type C strain JF 3721 contained 440 ng/mL CPB.
